# New live screening of plant-nematode interactions in the rhizosphere

**DOI:** 10.1038/s41598-017-18797-7

**Published:** 2018-01-23

**Authors:** Felicity E. O’Callaghan, Roberto A. Braga, Roy Neilson, Stuart A. MacFarlane, Lionel X. Dupuy

**Affiliations:** 1 The James Hutton Institute, Invergowrie, Dundee, DD2 5DA Scotland United Kingdom; 2 Federal University of Lavras, CP 3037, Lavras, MG 37.200-000 Brazil

## Abstract

Free living nematodes (FLN) are microscopic worms found in all soils. While many FLN species are beneficial to crops, some species cause significant damage by feeding on roots and vectoring viruses. With the planned legislative removal of traditionally used chemical treatments, identification of new ways to manage FLN populations has become a high priority. For this, more powerful screening systems are required to rapidly assess threats to crops and identify treatments efficiently. Here, we have developed new live assays for testing nematode responses to treatment by combining transparent soil microcosms, a new light sheet imaging technique termed Biospeckle Selective Plane Illumination Microscopy (BSPIM) for fast nematode detection, and Confocal Laser Scanning Microscopy for high resolution imaging. We show that BSPIM increased signal to noise ratios by up to 60 fold and allowed the automatic detection of FLN in transparent soil samples of 1.5 mL. Growing plant root systems were rapidly scanned for nematode abundance and activity, and FLN feeding behaviour and responses to chemical compounds observed in soil-like conditions. This approach could be used for direct monitoring of FLN activity either to develop new compounds that target economically damaging herbivorous nematodes or ensuring that beneficial species are not negatively impacted.

## Introduction

Free living nematodes (FLN) are microscopic worms that live in the soil. They move freely through soil making use of the thin film of water surrounding the soil particles. Although most soil nematodes are beneficial and play a role in the re-cycling of nutrients, herbivorous species of FLN can cause major damage to crops. Herbivorous FLN feed on plant roots by injecting a needle-like feeding structure known as a stylet into plant tissues. Besides making roots more susceptible to stress, the lesions give access to other harmful pests such as fungi or bacteria^1^. Certain species of herbivorous FLN also cause significant crop damage worldwide by transmitting viruses, e.g. tomato blackring virus and tobacco rattle virus (TRV) inducing spraing disease in potatoes. The deleterious impact of herbivorous nematodes on food security is likely to increase due to the rise in atmospheric temperatures increasing nematode reproduction rates^2^ and the reduction or withdrawal of established chemical controls in response to societal, environmental and health concerns eg. in the EU, the US and Australia^3^. This has led to a need for alternative control methods, such as incorporating naturally occurring plant defensive compounds into the soil^4,5^.

Behavioural studies of FLN are traditionally based on petri dishes containing gels or nematode growth medium. Agar has been routinely used to study nematode chemotaxis^6–11^. Gel or liquid media, however, lack the physical texture necessary for root growth and nematode movement similar to that in real soil, and as a result understanding the mode of action of plant-feeding pests is very difficult to assess. In addition, the density of the media presents difficulties when trying to regulate the concentrations of solutes and odorants and other gradients such as temperature and oxygen partial pressure^12^. The study of nematode behaviour could therefore benefit greatly from live imaging systems if detection could be made in a substrate that mimics soil. This has, however, remained challenging. Because of their small size (typically between 40 μm to 1.5 mm in length^13^) and mobility, studying FLN behaviour requires observations to be made both over a large region of the soil volume and with sufficient resolution to detect individual nematodes. Being composed of 70% water, nematodes are optically near transparent, have a cuticle practically impermeable to stains^14^, and live nematodes show little contrast to distinguish them from the other soil constituents. In addition, the opacity and heterogeneity of the soil matrix makes observations *in situ* extremely difficult.

Techniques based on X-ray computed tomography and magnetic resonance imaging^15,16^ have the potential to image through soil and assess root damage caused by herbivorous nematodes^17–19^, but none of these techniques are suitable to detect nematodes directly. Optics has remained the preferred approach in nematode studies but techniques such as Confocal Laser Scanning Microscopy^20^ or multi photon imaging^21,22^ rely heavily on fluorescent markers to produce image contrast. However, techniques for fluorescent labelling of nematodes are extremely limited. Fluorescently-labelled endoparasitic nematodes have been imaged *in planta* by confocal microscopy^23^, but there are no known vital stains that can be applied ubiquitously to FLN extracted from soil. Recently, light sheet based techniques such as SPIM (Selective Plane Illumination Microscopy) have shown great potential for live imaging studies^24–28^, but light scattering itself may not allow easy separation of nematode and root in the image^29^. On the other hand, techniques based on the dynamic light scattering created by living organisms, termed biospeckle, have shown potential as a simple, rapid and non-invasive means of tracking biological activity^30–32^. Biospeckle has the advantage of enhancing the contrast between living organisms from their abiotic background. This technique has however so far largely been used for the acquisition of 2D rather than volumetric data, and to monitor bioactivity within homogenous media such as air or gel.

Despite the recent technological progress in imaging some nematodes there is as yet a lack of efficient approaches that allow the simultaneous assessment of chemical treatment and nematode behaviour in the rhizosphere. A substrate is required which is transparent enough for light penetration, provides the physical environment for realistic root growth and nematode burrowing behaviour, as well as being able to mimic the diffusion of fumigants in soil. In particular, FLN require water and air filled pores and channels within the soil to move around by using the films of water adhering to the soil particles^33–38^. In this study, we have built on recent advances in light sheet microscopy, biospeckle laser imaging and new transparent soil techniques^39^ to streamline analyses of nematode activity in the rhizosphere. We have assessed the ability of various techniques to 1) culture plants and nematodes in soil-like conditions while allowing easy introduction of chemical controls; 2) rapidly locate and map the distribution of live nematodes in the rhizosphere of a growing root in 3D; 3) quantify infestation levels; and 4) image nematode behaviour in high resolution. Finally, we propose a general framework to assess FLN risk to plant health and test the efficacy of chemical controls.

## Methods

### Plant and nematode material

Plants were grown from seed: Lettuce, (*Lactuca sativa*), tobacco (*Nicotiana tabacum* and *N*. *benthamiana*), thale cress (*Arabidopsis thaliana*) and petunia (*Petunia multiflora*). Seeds were surface sterilized by washing in 10% bleach for 20 minutes and then washed 5 times in sterile dH_2_O. Planting in transparent soil was carried out under sterile conditions. Plants were grown at 25 °C in light cycles of 16 hours light to 8 hours dark. Water was periodically added to the growth chambers to prevent desiccation. The “soil” region was kept shaded from light by covering it with tinfoil. Mixed assemblages of FLN were extracted from local soil (56.46° N, 3.06° W) using a modified sieving and decanting technique^40^.

### Transparent soil

Transparent soil was prepared from Nafion® precursor beads (Ion Power Inc., USA) largely as previously described in Downie, *et al*.^39^. The procedure was modified to improve milling and the cleaning of the soil particles for re-use. Nafion® pellets were first fractured in a cryogenic mill (SPEX SamplePrep 6770) to sizes of 250–1250 µm. Samples were left in the liquid nitrogen for at least 30 minutes prior to the milling process. Particles were converted to anionic form by washing in 6 M KOH, 35% 5 M DMSO at 80 °C and subsequently in 3 M nitric acid at room temperature. Murashige and Skoog Basal Medium (Sigma M5519) at 4.4 gL^−1^ was used to titrate Nafion® particles with minerals and to raise their pH to 6–7. Before planting, transparent soil was sterilized by autoclaving for 20 minutes at 121 °C. For re-use, the particles were washed in 1 M sulphuric acid and 1 M H_2_O_2_ at 65 °C for the complete removal of organic residues. The whole chemical treatment was repeated multiple times, if necessary, and until complete transparency of the soil particles was achieved.

### Microcosms for nematode observation

Purpose-built culture chambers were developed to allow for both plants and nematodes to be cultured within a transparent soil microcosm. The chambers consisted of two microscope slides which were separated by either nitrile (RS components 683–0771 and Cromwell BS330) or silicone (Polymax BS331SR70) spacers and the two slides held together by rubber bands of parafilm film. Spacers were either ring-shaped or open on the top side, with the opening covered with plastic paraffin film (Cole- Parmer PM996 Wrap). Nematodes and liquid solutions were injected with a syringe through the spacer. Alternatively, spacers open at the top admitted more light to the plant and allowed nematodes to be inserted by hand using a dissecting needle at chosen locations.

### Refractive index matching

Nafion® has a refractive index of 1.348, which is slightly higher than that of water (1.330). To obtain transparency we examined a solution of a high molecular weight sugar, trehalose, as well as two silica suspensions, polyvinylpyrrolidone coated silica (Percoll®; GE Healthcare 17-0891-01) and Ludox® TMA (Sigma 420859) for suitability as refractive index matching liquids. Percoll® is routinely used as a centrifugation medium in the extraction of live nematodes, we have also tested Ludox® TMA as an alternative^41,42^. Matching of the refractive index was carried out using a laser light sheet passing through samples of transparent soil saturated with trehalose solutions. Projection images of the sheet were taken before and after passing through each sample, the former images subtracted from the latter, and the total intensity of the resulting image calculated. At optimal refractive index, light scattering was at its lowest, resulting in the lowest intensity of the subtracted images. Intensity was measured in grey values on ImageJ after converting images to 8-bit. Grey values represent the brightness of the image pixels on a scale from 0 (black) to 255 (white). To compare refractive index matching between water, trehalose, Percoll® and Ludox®, Nafion saturated with each liquid was also filled into fluorimeter cuvettes of 1 × 1 cm^2^ in cross section. A black line was drawn behind each cuvette. Transparency was assessed by measuring the grey values across each sample, perpendicularly to the black line. Transparency was greatest where the difference between black line and background values was greatest. Four cross sections were made for each sample. In microcosm experiments, the refractive index matching liquid was injected into microcosms slowly from below to allow air bubbles to escape. To remove air bubbles, a pump (12 V vacuum pump; RelChron PPROB-10398) was attached to a needle perforating the ring-shaped spacer of the culture chamber; air was pumped out of each chamber before and after inserting the refractive index matching liquid. Afterwards samples were left to stand vertically for at least 12 hours while the nematodes burrowed into the soil and during which time air bubbles were allowed to escape.

### Staining

Detecting nematodes in transparent soil is difficult because of their small size in comparison to the volume of the samples to be examined. Therefore, we have investigated the use of dyes to increase the contrast in microscopy images. Sulforhodamine B (Sigma S1402) at 1 mg L^−1^ was used to stain soil particles as was previously demonstrated by Downie, *et al*.^39^. Roots were stained with calcofluor (fluorescent brightener 28; Sigma F3543) as described by^43^ at 5–10 mg 100 ml^−1^. There is no known stain for FLN. The approach for staining FLN was, therefore, to identify a biocompatible stain with potential for being ingested by nematodes during feeding. 12 mM fluorescein diacetate (Sigma F7378) was diluted 1:20 in ethanol and then further diluted 1:20 in sterile water. Stains were either added diluted in water, injected into the microcosm through the spacer and then drained before adding the refractive index matching liquid, or dissolved directly into refractive index matching liquid before imaging. For sulforhodamine B stains, a laser excitation of 561 nm and an emission filter of 595 nm were used. 405 nm and 488 nm excitation wavelengths were used for calcofluor and fluorescein (emission wavelengths of 450 nm and 515 nm respectively).

### BSPIM for macroscale imaging of soil microcosms

Because staining of FLN is challenging, we have investigated label-free approaches to detect nematodes. An imaging system was developed to capture image data from nematode activity without the need for fluorescent markers. The new method, termed Biospeckle Selective Plane Illumination Microscopy (BSPIM), combines the principle of Selective Plane Illumination Microscopy (SPIM) with an analysis of the dynamics of the light scattering of the biological sample (called Biospeckle Laser Imaging or BSL). Biospeckle Laser Imaging uses the speckle patterns created by lasers when illuminating a scattering medium. Lasers produce in-phase, known as coherent, rays of light. When these are shone through materials their constituent elements act as scatterers and alter the path and phase of the light rays returning to the observer^44,45^. This creates interference patterns known as speckle. In biological samples, heterogeneities, such as cells and their components vary their shapes or locations with time. The time dependent speckle patterns are termed biospeckle and can be analysed to create images of biospeckle activity by means of graphical or numerical biospeckle indexes. Illumination from a SPIM system consists of a thin sheet of light passing through the sample. SPIM allows optical sectioning by only illuminating the sample within the focal plane. It is used for quick *in vivo* scanning of biological samples while keeping photobleaching to a minimum^24,25,46–48^. Light in a SPIM system remains coherent and therefore scattering of the biological tissue produces interference patterns that can be analysed using methods developed for regular biospeckle analyses.

**Figure 1:**
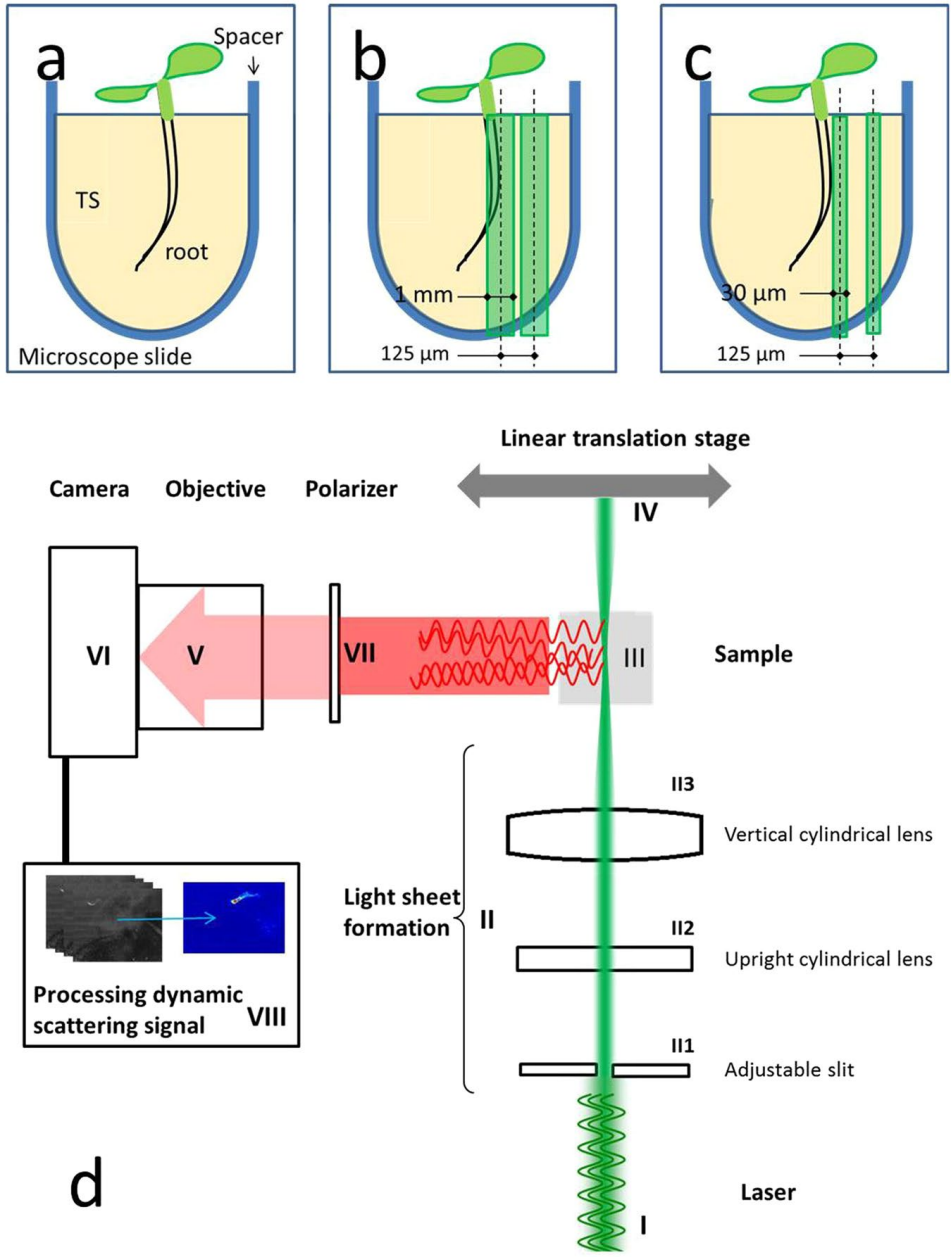
Imaging systems for the detection of nematode activity. (**a**) Plants and nematodes cultured in chambers created by two microscope slides separated by a spacer, between which transparent soil (TS) was held as the growth medium. The chambers were scanned by a laser light sheet either to detect the numbers of nematodes present or to observe their activity within the rhizosphere. Two scanning modes were used: (**b**) nematode automated detection was carried out using a light sheet of 1 mm in width moved by increments of 125 µm (an area of overlap, not shown, is created which prevents small or mobile nematodes being missed). (**c**) 3D rendering was carried out with light sheets of 30 µm in width by increments of 125 µm. (**d**) Principle of Biospeckle Selective Plane Illumination Microscopy (BSPIM) Imaging. The illumination is composed of a laser light source and two cylindrical lenses. Light from a laser (I) is first passed through an adjustable slit (II1) before being vertically broadened by a cylindrical lens (II2) to produce the light sheet. A second cylindrical lens (II3) is used to focus the light sheet on the sample (III), which is translated along the z-axis by a motorised stage (IV). Images were collected by stereomicroscope and camera (V-VI) perpendicularly to the sheet of light while using a polariser (VII) to remove reflected light. Image sequences were subsequently analysed for dynamic scattering (VIII) resulting in a map of biospeckle intensity for each step moved by the sample.

An imaging system was developed so that the chambers in which plants and nematodes had been cultured could be scanned for biospeckle activity, in order to detect nematodes and quantify their activity in soil-like conditions (Fig. 1a–c). The imaging system (Fig. 1d) consists of a green laser diode module (CPS520, Thorlabs) of 420 nm wavelength and 4.5 mW intensity (I). The laser is formed into a light sheet by a series of steps (II) in which it is first passed through an adjustable slit (Thorlabs VA100/M), and a cylindrical lens (Thorlabs LJ1878L2) is then used to expand the beam vertically to produce a sheet of light 55 mm in height at the point where it reaches the sample. The sheet of light passes through a second cylindrical lens (Thorlabs LJ1640L1) to focus the sheet of light in the horizontal plane and is placed in the system so that the thinnest section of the sheet (30 µm) is at the intersection with the z-axis of the microscope. The sample (III) was held in place with a STANDA 4SCML-2 self-centering lens mount with silicon tubing fitted on the holding pins for improved grip of the sample; and a STANDA 8MT167 motorised linear translation stage (IV) was used to control the movement of the sample for adjustment and scanning. The optics for image acquisition consisted of a Leica MZ16 FA stereomicroscope fitted with a 0.5 × plan achromatic objective with 135 mm working distance (V), and a Leica DFC350FX camera (VI). A 90° angle mirror was placed below the objective so that plant samples could be held vertically. A polarising filter (VII) was placed in front of the objective to limit noise from reflected light. The laser diode module was set horizontally at right angles to the z-axis so that intersection of the beam axis with the z-axis takes place in the plane of focus and in the centre of the field of view. Image acquisition was paused for 0.4 seconds after moving the stage. 3D volume scans of samples were acquired by moving the stage by increments of 125 µm and repeating the measurements for each new position of linear translation.

### Software and image analysis

Software tools allowing stage movement and image acquisition were developed in C++ using Application Programming Interfaces (APIs) provided by the hardware manufacturers (SMCDLL.dll for Standa components and FxLib.dll for the DFC350FX camera) and a python wrapper was built for the graphical user interface. For each position of the linear translation stage, images were acquired at 10 fps and acquisition was stopped after 64 images were collected.

For each position of the stage, an image that maps the activity of biospeckle was obtained using the GD (generalized differences) method described by Arizaga, *et al*.^49^ using the following formula,

1 $$GD=\sum _{i}\sum _{j}|{I}_{i+j}-{I}_{i}|$$

where *I*_*i*_ is the pixel intensity at a given position at the *i*^*th*^ image and *j* is the time increment between two images in the stack. The GD transformation therefore provides a single image that maps the total variation in pixel intensity within the stack of brightfield images at a given depth within the sample. Scripts^50^ were developed to reconstruct the BSPIM data automatically by calculating the biospeckle intensity from each time lapse sequence recorded at each position of the sheet within the sample. One GD image was constructed from 64 bright field images. The time lag between two successive GD images including the motion between motorised stage positions was 6.8 seconds.

A signal to noise ratio metric (SN) was used to assess the quality of the contrast produced by BSPIM imaging. Signal to noise ratio metric, used for example in Magnetic Resonance Imaging^51^, compares the increase in signal intensity (here the pixel value at the location of nematode activity) with the standard deviation of the pixel intensity of the image. Here we use the following metric to characterise the signal to noise ratio

2 $$SN=\frac{{I}_{s}-\langle I\rangle }{\sigma }$$

SN was estimated from data taken along a transect from the side of the sample, through the transparent soil matrix and the nematode. *I*_*s*_ is the pixel intensity of the detected object and *I* is the average intensity of the pixels along the transect. *σ* is the standard deviation of the pixel intensity along the transect.

Automatic detection of nematodes was carried out with segmentation of 3D image data (a flowchart and the script of the process can be found as Supplementary Material S1 and S2 respectively). Segmentation was implemented in FIJI software. Images were first cropped to a region of interest in the fluorescence cuvette; this either encompassed the Ludox® TMA-water mixing layer (0.8 mL) or a column of transparent soil saturated with Ludox® TMA (1.5 mL). Stacked images of GD reconstructed image data were converted to 8-bit images. Images were then filtered for long range variations (subtraction of boxcar average of radius 2 mm) and short range variations using a 3D Gaussian filter (x, y and z sigma all set at 80 µm). The 3D object counter plugin^52^ was then used to detect areas of biospeckle above an arbitrary brightness threshold of 40 and voxel size of 50 to filter out noise below the minimum speckle intensity and size of FLN. The threshold was applied consistently to all images for Ludox® TMA and Ludox® TMA plus transparent soil samples.

### Laser Scanning Confocal Microscopy for high resolution imaging of soil microcosms

Gaining insight into nematode feeding behaviour at the surface of the root requires high resolution imaging. Therefore, protocols of Confocal Laser Scanning Microscopy (CLSM) for plant nematode co-cultures were developed. Confocal imaging was carried out on a Nikon A1R confocal laser scanning microscope using either x2, x4 or x10 magnification. The lasers were an Argon laser for 488 nm excitation, a sapphire laser for 561 nm and a violet diode for 405 nm; these were used in combination with emission filters of 515/30, 595/50 and 420/50 nm wavelengths, respectively. Image sequences were acquired by using open loop continual recording while using a resonant scanner for rapid image capture. 3D projections of confocal z-stacks were created on Nikon NIS- ElementsViewer 4.20.

### Experiment 1 – biocompatibility of new transparent soil microcosms

Nafion® is a polymer tetrafluoroethylene of the Teflon™ family. Like most Teflon™ polymers, Nafion® is chemically inert (in its acidic form), and when adjusted at the correct pH it provides a suitable environment for soil biological studies^39^. Making the soil transparent, however, requires adding liquid solutions to the Nafion® fabric, and these liquids can affect biological processes drastically. Therefore, the first experiment focused on testing whether the new refractive index matching liquid solutions affected plants. Lettuce seeds were sown separately in cuvettes containing 500 mg of transparent soil filled with 0.75 mL of either sterile water, Ludox® TMA, Percoll® or trehalose with refractive indices adjusted to 1.348. Cuvettes were capped with plastic paraffin film (Cole- Parmer PM996 Wrap) to prevent desiccation and incubated at 25 °C at 16 hrs light: 8 hrs dark. Seed germination rate was recorded after 1 week. 20 seeds were sown per treatment.

The activity of soil FLN in the different refractive index matching liquids was also investigated using a visual assessment termed the spontaneous movement approach. Motility is required by FLN for migration, mating and infection and spontaneous movement has proven to be a reliable measure of nematode survival^53–57^. The spontaneous movement approach consists of scoring nematodes for visible motor activity under bright field illumination. 300 mg of transparent soil were immersed in a 0.1 mL solution of Ludox® TMA, Trehalose or Percoll® diluted to a refractive index of 1.348. Nematodes were freshly extracted from soil before the experiment and only those individuals having demonstrated spontaneous movement before their transfer into the microcosms were used for the experiments. Nematodes were transferred to microcosms by handpicking. The experiment consisted of 5 microcosms each containing 5 nematodes. Spontaneous movement of nematodes viewed under x 5 magnification objective was recorded 24 hours, 1, 2 and 5 weeks after their insertion into the microcosm chamber. Microcosms were kept at 4 °C between observations.

### Experiment 2 – suitability of transparent soil microcosms to study responses to chemical treatment

The microcosms were tested for their suitability to study nematode responses to nematicidal compounds. Dazomet (Pestanal®, Sigma 45419) was chosen in this experiment because it releases methyl isothiocyanate (MITC). MITC is a broad-spectrum fumigant used against nematodes, fungi, insects and weeds^58^. It pervades soil mainly by diffusing through water^59,60^. The pesticide was applied by dissolving 100 mg L^−1^ of dazomet in Ludox® TMA solution adjusted to a refractive index of 1.348. 300 mg of transparent soil were immersed into 0.1 ml of the dazomet / Ludox® TMA solution. Spontaneous movement of added nematodes was recorded as described for experiment 1.

### Experiment 3 – automatic detection of nematode activity

In the third experiment, we tested whether automatic detection of nematodes can be achieved in microcosm experiments. Due to the lack of ubiquitous staining procedures for FLN, BSPIM was used to assess the feasibility of the method. Nematodes were freshly extracted from soil before the start of the experiment and those individuals used showed spontaneous movement before their transfer into the cuvettes. Firstly, nematodes were inserted into fluorimeter cuvettes (Sigma C0918) filled with 1 mL Ludox® TMA with a refractive index of 1.357 before adding 1 mL of water. After 10 minutes, a density gradient was formed due to the sedimentation of silica nanoparticles contained in the solution. The density gradient ensures that nematodes are located at the midpoint of the cuvette. Secondly, to test nematode detection in transparent soil, cuvettes were filled with 1.5 mL of transparent soil saturated with Ludox® TMA with a refractive index of 1.349. The experiment consisted of cuvettes each containing between 0 to 20 nematodes in 3 replicates for tests in Ludox® TMA, and 5 for tests in transparent soil + Ludox® TMA. To investigate BSPIM as a method that can reliably distinguish live from dead nematodes, we compared the counts of detected heat killed nematodes with those of detected freshly extracted, live nematodes. The experiment was therefore repeated with nematodes that had been killed by exposure to 60 °C for 90 seconds prior to their insertion in Ludox® TMA. Heat of 60 °C has previously been used as a reliable method of killing nematodes resulting in them forming a consistent habitus or shape thus having negligible effects on the surface area and optical density of the nematodes^61^. Imaging was carried out with the BSPIM system detailed above. In this experiment however, the cylindrical lens used for focusing (II3 in Fig. 1) was removed to generate light sheets with increased thickness (1 mm) to allow for faster scanning and overlapping of the optical sectioning. The linear translation stage was moved by increments of 125 µm and covered the entire depth of the cuvette (1 cm), and 64 images were recorded at each of the 80 positions along the depth of the cuvette. Automatic detection of nematodes was then obtained by 3D segmentation techniques.

### Experiment 4 – detection of nematode activity in the rhizosphere

In the final experiment, we assessed the possibility to study the behaviour of nematodes in the rhizosphere where conditions approach those found in real soil. Experiments were conducted on 5 samples in fluorimeter cuvettes containing 2.5 g transparent soil and 5 samples in open sided microcosm chambers containing 300 mg transparent soil. The refractive index of the transparent soil was matched with Percoll® solutions. Lettuce seeds were sown directly into transparent soil and grown for 1 week at 25 °C in light cycles of 16 hours light to 8 hours dark. Nematodes that showed spontaneous movement were then transferred into the chambers. Prior to analysis, plants and nematodes were left for a further 1–2 days at 25 °C in light cycles of 16 hours light to 8 hours dark with the root system in the dark.

First, the system was used to characterise the contrast produced by the biospeckle data. Because roots also exhibit strong levels of biospeckle activity^44^, the unfiltered biospeckle signal would not be suitable to distinguish nematodes from roots. However, roots are much less transparent than nematodes and show higher pixel intensity. Therefore, the root signal was filtered by adjusting the exposure of the camera in order to saturate the intensity of pixels corresponding to roots. Higher exposure time reduced the contrast between root and background while keeping the contrast of nematodes very high. Biospeckle intensity data were then compared with bright field images. Analysis of biospeckle activity was carried out using the BSPIM system detailed above. No stains were used for biospeckle imaging.

Samples were also used to obtain high resolution images of nematode behaviour around their feeding sites. For this, microcosms constructed from microscope slides were used which maximised the field of view for confocal laser scanning microscopy. 15 soil microcosm systems were constructed with spacers open at the top to allow in more light and reduce mechanical stresses to the plant. Lettuce seeds were germinated on 5 gL^−1^ MS phytagel and then transferred into microcosm chambers. Chambers contained 6 g of transparent soil. To gain contrast between plants, soil and nematodes for high resolution imaging we either used stains or autofluorescence, as described above.

### Data availability

The datasets generated and analysed during the current study are available from the corresponding author on request.

## Results

### Development of a model system for the study of FLN responses to chemical compounds

**Figure 2:**
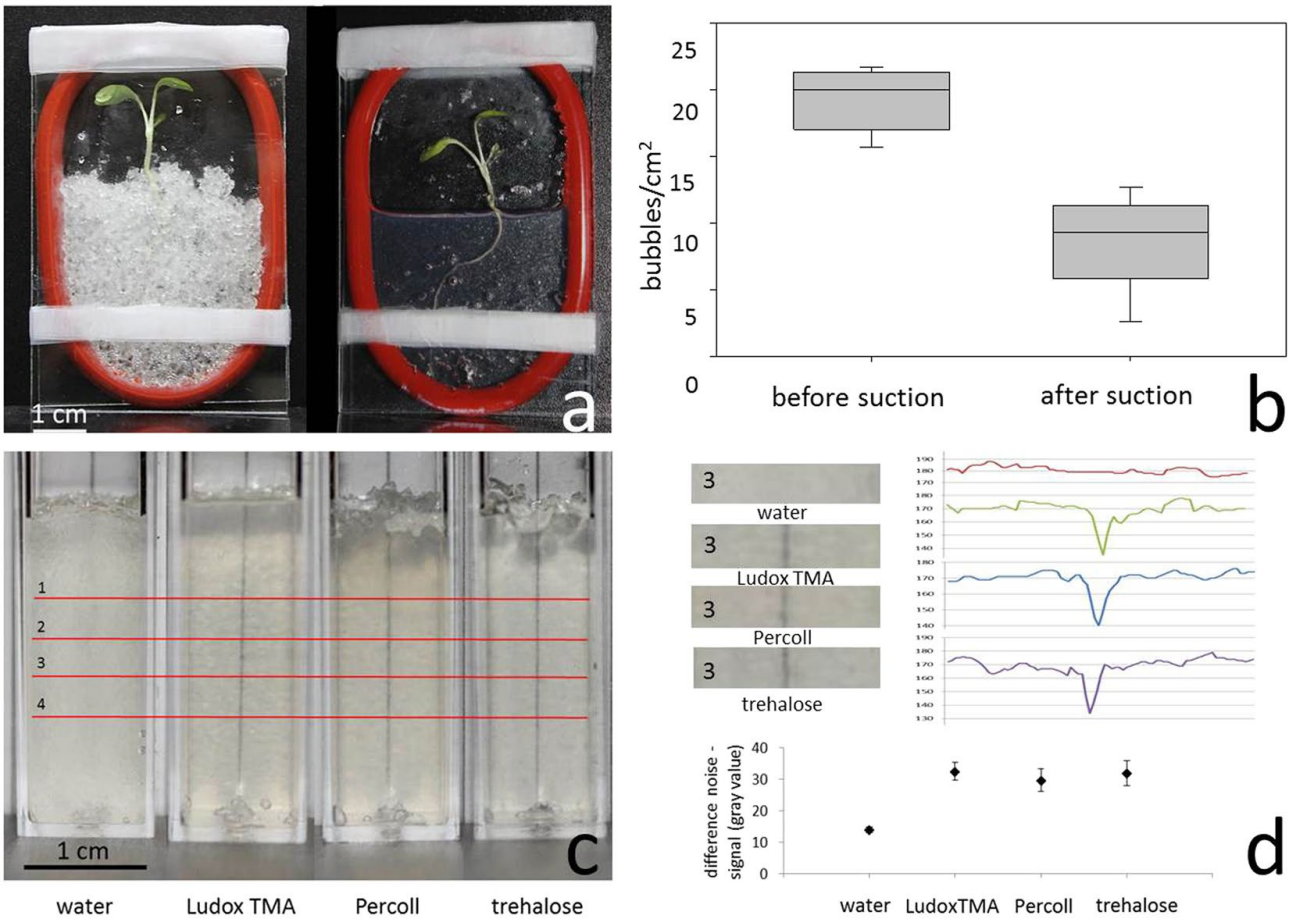
Optimisation of transparent soil microcosms. (**a**) Lettuce grown for 8 days in transparent soil growth chambers consisting of two double-size microscope slides separated by a silicone ring. Plants are shown before and after immersion in refractive index matched Ludox® TMA. (**b**) Bubbles were removed from saturated transparent soil by suction, resulting in a significant decrease in the number of bubbles per cm^2^. (**c**) Fluorimeter cuvettes filled with transparent soil saturated in water and refractive index matched Ludox® TMA, Percoll® and trehalose. A line has been drawn behind the cuvettes for comparison of optical clarity. (**d**) Quantitative assessment of optical clarity of the substrates shown in (**c**). Measurements were made along 4 cross sections; these ran perpendicularly to the black line behind each sample and are shown in red in (**c**). Top left, close-up view of the samples at cross section 3. Top right, grey values of the image along cross section 3. Because grey values have black as their lowest intensity, grey values drop at the point where cross sections pass the black line. As the black line is clearer the better the refractive indices are matched, a larger drop in grey values indicates greater transparency. Below are shown the mean difference between grey values for noise and signal (i.e. the minimum grey value). Values are averaged across the 4 sections and show that refractive index matching with the colloids Ludox® TMA and Percoll® achieved similar transparency to that of the trehalose solution.

We present a new system that overcomes many of the limitations of current techniques for nematode observation in soil (Fig. 2). The system is based on artificial transparent soil as a substrate for plant growth and nematode behavioural studies. The transparent soil mimics the physical and chemical properties of natural soil, with the ability to make live observations and quantitative imaging of nematode activity within the rhizosphere. The transparent soil is placed in sealed or semi-sealed chambers where plants, nematodes and other micro-organisms can grow for several weeks (Fig. 2a). Because the chamber design is simple, contains few parts and is free of adhesive, it can be sterilised and reused. The chambers use generic materials and components to create a modular and inexpensive system where the size and number of microcosms can easily be adapted to a range of different experiments. The joints between the observation windows are made of soft elastic material (nitrile or silicon) and precise control of the environmental conditions during the experiment is ensured through gas and liquid exchanges with the microcosm. Experiments carried out throughout this study showed that liquids can easily be exchanged by injection and suction through the nitrile and silicone spacers, with the elasticity of the spacers preventing leakage once needles were withdrawn. Injections were used for the introduction of nematodes, stains, liquids for refractive index matching, nematicides and nutrients. Injection can also be used to control the atmospheric pressure or for introduction of other gases such as CO_2_ or fumigants. Finally, the use of glass plates was designed to allow both upright imaging of the roots with light sheet or biospeckle, but also in a horizontal position for use in confocal laser scanning microscopy.

The microcosm system was optimised for transparency. Removal of air was achieved during the filling of the transparent soil with the matching liquid. Applying a mild vacuum approximately halved the number of bubbles per cm^2^ trapped within the soil and matching liquid (Fig. 2b; paired t-test, p = 0.009; average of 3 readings for n = 5). In long term experiments in which Nafion® and matching liquid were left for a week or more, the nitrile spacers were found to slightly discolour the transparent soil particles. Silicone spacers however, did not produce discoloration in any experiment (up to 16 days) and were therefore preferred for long term experiments, carried out by light microscope or CLSM. Biospeckle measurements were carried out within 24 hours of inserting nematodes into freshly prepared microcosms, ruling out any discoloration by nitrile. Adjustment of the refractive index was achieved using a sugar (trehalose) and two different colloidal suspensions (Ludox® TMA (pH 6) or Percoll®). Solutions of sugars and sugar alcohols were used in previous studies^39,62–65^ because they are inexpensive and highly transparent when in solution. Here we have tested trehalose because it has a higher molecular weight than the common sugars, e.g. glucose or sorbitol, thereby increasing the refractive index per mole ratio. Colloids are suspensions of nanoparticles that have a limited effect on the osmotic potential of the solution. Silica colloids have less impact on plants and micro-organisms because they are found in natural soils, but they are less efficient at transmitting light. Optimal matching of the refractive index of Nafion® was found for liquids with a refractive index of 1.346–1.348. However, because gradients in the refractive index form with time, solutions with a refractive index of up to 1.349 were found to improve the transparency of the upper part of the sample. The two colloids Percoll® and Ludox® TMA attained refractive index matching of Nafion® similar to that of the trehalose sugar solution (Fig. 2c). All three were significantly more transparent than water (Fig. 2d), but there was no statistically significant difference in transparency between trehalose, Ludox® TMA and Percoll® (1-way ANOVA and post-hoc LSD, p < 0.001, n = 4).

### Bio-compatibility of transparent soil systems (experiments 1 and 2)

**Figure 3:**
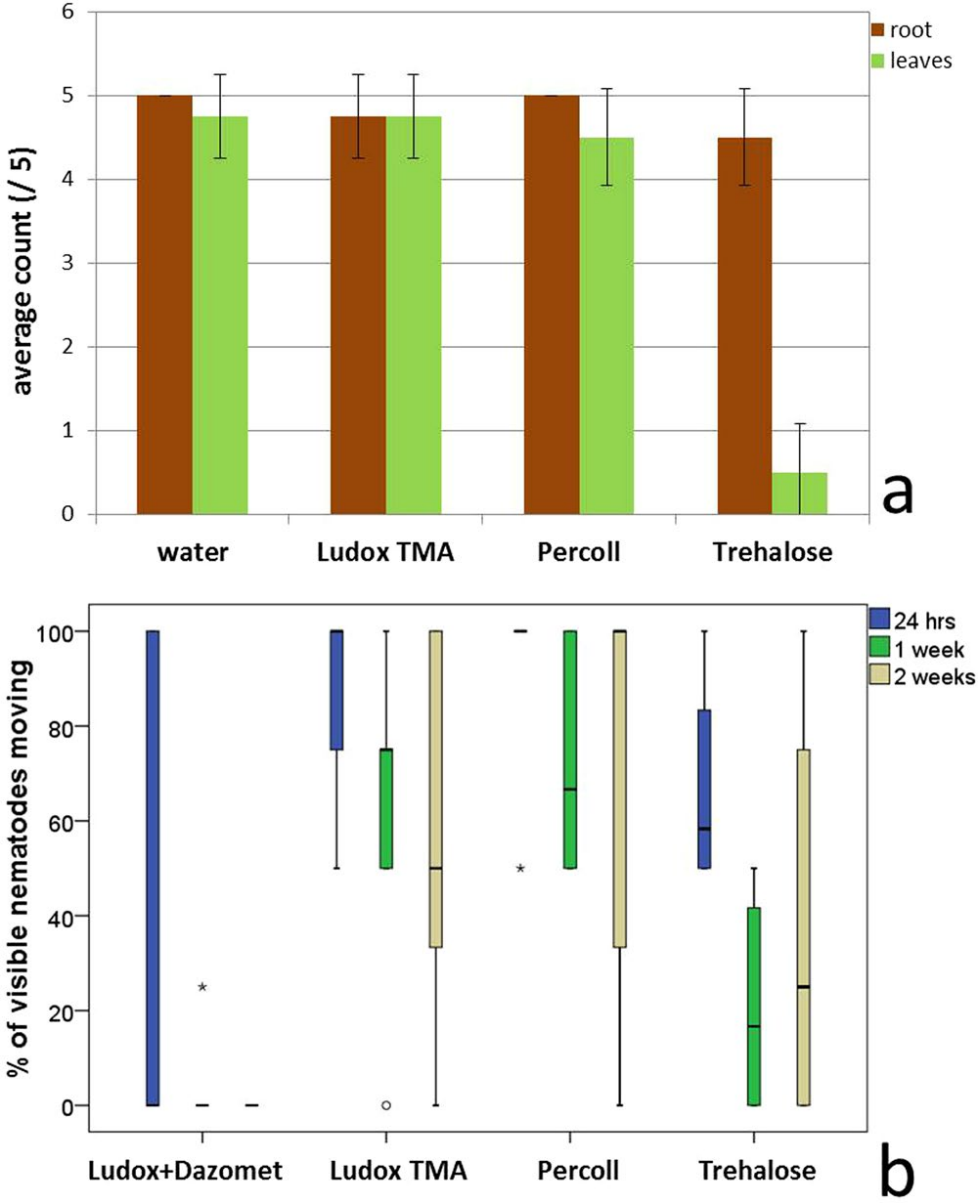
Nematode motility and plant germination rate. (**a**) Comparison of refractive index matching liquids Ludox® TMA, Percoll® and trehalose with water as a germination and growth medium for plants in transparent soil. Columns represent the average count within replicate groups of 5, with error bars representing the standard deviation. (**b**) Survival of soil FLN in transparent soil with Percoll®, Ludox® TMA, trehalose and dazomet dissolved in Ludox® TMA at 100 mg L^−1^. Each treatment was tested on 5 microcosms of transparent soil containing 5 nematodes each. For each microcosm, the percentage of moving nematodes was calculated as a percentage of all visible nematodes. The figure shows the range of percentages across all 5 microcosms, with boxes representing 50% of observations and whiskers representing x 1.5 the interquartile range. Outliers outside x 1.5 and 3 the interquartile range are represented by an open circle and asterisks respectively. Spontaneous movement over the first 24 hours was visible in all samples for Percoll®, Ludox® TMA and trehalose but not in the presence of dazomet. After 1 week, less activity was also observed in trehalose. Spontaneous movement declined in the presence of dazomet within 24 hours and was completely absent after 2 weeks.

All plant species studied in this paper (lettuce, thale cress, tobacco and petunia) grew for two weeks in transparent soil with water and produced root systems of several cm in length with lateral roots and root hairs. In experiment 1, we have tested the effect of the refractive index matching solutions on germination rate and root growth of lettuce seed. When the soil solution contained water, Ludox® TMA (pH 6) or Percoll® (pH 9), more than 90% of lettuce seeds had developed roots and leaves one week after sowing (Fig. 3a). Although the seeds had a slightly higher germination rate in the water control treatment, there was no significant difference between treatments (Fisher’s exact test, p = 0.487, n = 20). We did not notice differences in the size and morphology of the root systems of the lettuce plants grown in water, Ludox® TMA or Percoll®. Seeds sown in trehalose, however, turned brown shortly after germination and in 90% of cases failed to develop leaves (Fisher’s exact test, p = 0, n = 20).

In experiment 2, the survival of freshly extracted nematodes in refractive index matching solutions was assessed over a duration of 5 weeks. During the first 2 weeks, the spontaneous movement of nematodes remained high in transparent soil with either Ludox® TMA or Percoll®, with on average more than 60% of visible nematodes showing spontaneous movement (Fig. 3b). In the presence of trehalose, a strong decline in the number of nematodes showing spontaneous movement was observed (37% after 1 week) although this recovered slightly in week 2. In Ludox® TMA or Percoll® some decline in nematode spontaneous movement occurred after 5 weeks, however, spontaneous movements were still observed despite the absence of nutrient sources (data not shown). No activity was observed in Ludox® TMA with 100 mg L^−1^ dissolved dazomet after 2 weeks (significantly different from observations in Ludox® TMA alone, Fisher’s exact test, p = 0.0476, n = 5). This confirmed that pesticides can be successfully applied to our transparent soil microcosms. Nematodes were clearly visible with light microscopy, showing that the method of diluting pesticide within the refractive index matching liquid did not compromise clarity.

### Automatic detection of nematode activity (experiment 3)

**Figure 4:**
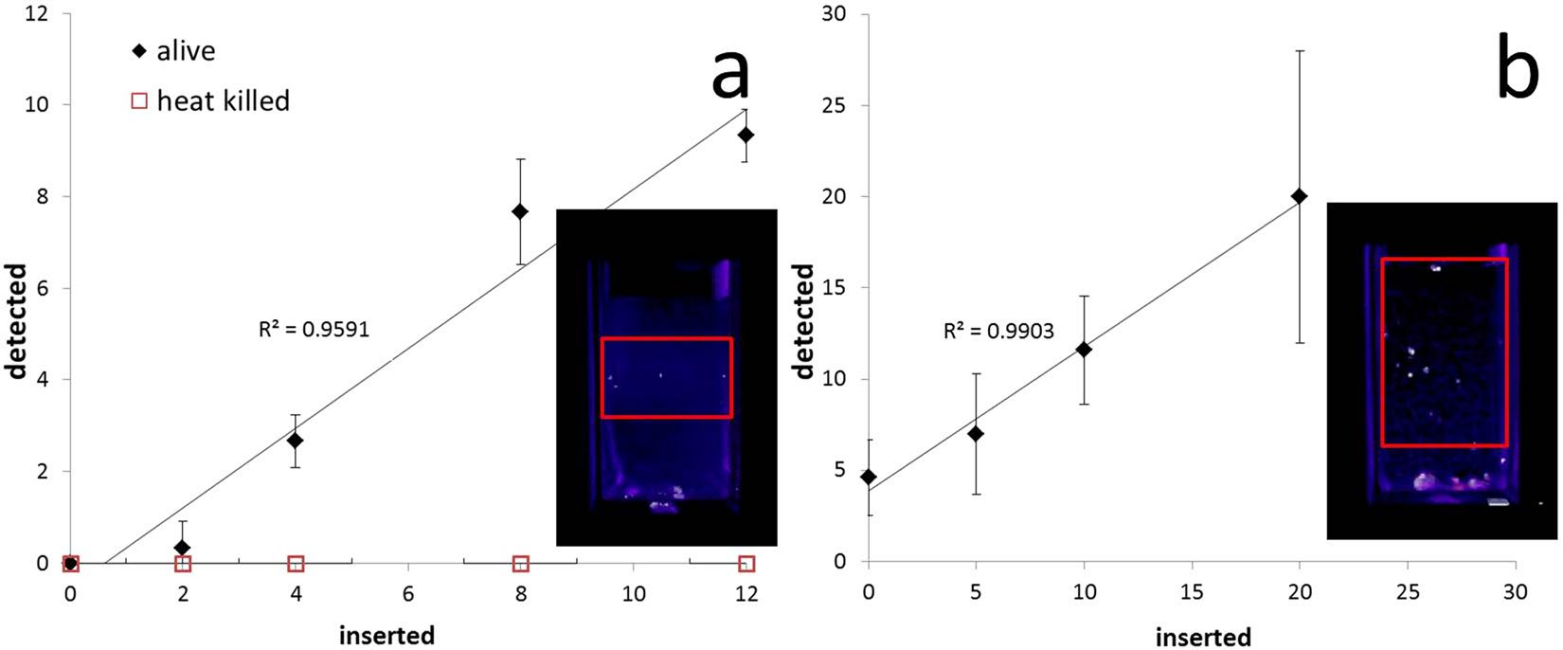
Correlations of numbers of inserted nematodes with mean numbers of nematodes detected by biospeckle, with 3 replicates for each data point and error bars representing the standard deviation. Images of biospeckle activity were obtained at different depth within the sample using the GD method. Images obtained with the GD method at different depths were stacked to constitute a volume image. The volume image was then filtered, and the number of areas with high activity detected automatically using ImageJ 3D object counter. (**a**) Nematodes detected while in suspensionin a Ludox® TMA solution. Black markers represent live nematodes, open red markers represent nematodes killed by 90 seconds at 60 °C. (**b**) nematodes detected in transparent soil immersed in Ludox® TMA with 5 replicates for each data point and the standard deviation represented by the error bars. Red boxes show the region of interest containing the nematodes.

In order to accelerate the discovery of chemical compounds for the management of FLN, it is important to design fast assays where activity of nematodes can be monitored in response to treatment. With samples prepared in standard 1 cm^2^ fluorimeter cuvettes, it was possible to automatically determine the number of living nematodes. The detection is based on the collection of BSPIM image data and use of 3D image segmentation to identify and count regions of high biospeckle laser intensity (an example of a BSPIM image sequence in Ludox® TMA can be found in Supplementary Video S3). The numbers of live nematodes inserted in the fluorimeter cuvettes at the start of the experiment were correlated with the number of detected objects in the GD image sequence (linear regression R^2^ = 0.9591; Spearman rank-order correlation, p < 0.001, n = 3) using the detection algorithm. However, when heat killed nematodes were inserted, the detection algorithm did not identify any object in the image (Fig. 4a). The experiment was repeated in transparent soil volumes of 1.5 mL (Supplementary Video S4 gives an example). Images collected from transparent soil samples had an increased level of noise because of the heterogeneity of the refractive index of the solution (Ludox® TMA). Nevertheless, a strong correlation was again observed when examining numbers of FLN varying from 0 to 20. The correlation between the number of nematodes inserted and the number of nematodes detected by BSPIM was highly significant (linear regression R^2^ = 0.9903; Spearman rank-order correlation p < 0.001, n = 5; Fig. 4b).

### Detection of nematode activity in the rhizosphere(experiment 4)

Understanding how nematodes move, feed and respond to physical and environmental cues is challenging. This involves (1) determining the location of the nematode with regard to plant roots and soil depth; (2) identifying the species of the detected nematode; (3) measuring a number of biological traits such as size, age and sex (4); measuring anatomical features of plant roots, such as the presence of root hairs, the maturity of the root tissue; and (5) determining the mobility and vitality of each individual nematode e.g. alive and active, alive and inactive, dead. Measurements must be made on nematodes that are typically only 40–1500 µm in length and often under 30 µm in width and in a soil volume of only several mL.

**Figure 5:**
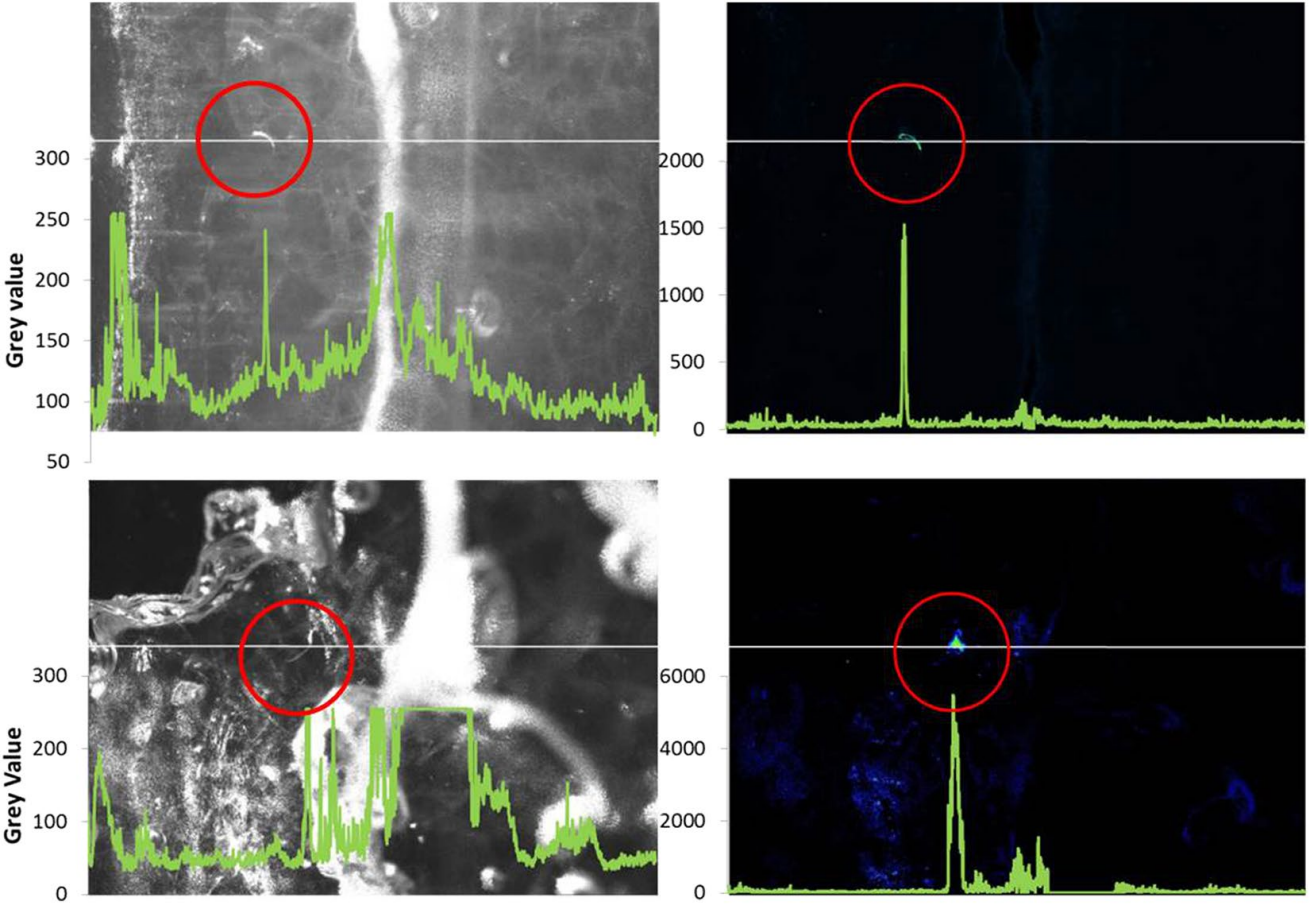
Two examples of bright field images (on the left) and images of the biospeckle activity obtained using the GD method (right) of FLN in the vicinity of lettuce roots grown in transparent soil lit by a green laser light sheet. Biospeckle from the root has been blotted out by saturation, allowing nematodes (circled in red) to be easily located. In comparison to bright field, biospeckle imaging provides enhanced contrast and the reduction of noise from scatter, visible as white pixels in the brightfield images. Superimposed green traces show the grey values of cross-sections through the images, shown as a white line. Grey values for bright field images had higher levels of variation caused by the scatter of laser light by the transparent soil environment and the root, visible here only in the bright field images. By comparison, this variation was greatly reduced in the biospeckle images.

In experiment 4 we showed it is possible to combine macro scale detection of nematodes using BSPIM with high resolution imaging using confocal laser scanning microscopy. Biospeckle Laser Imaging was the most effective detection tool as it used large fields of view (5 cm^2^) to scan extensive portions of the root architecture which reached depths of up to 5 cm. In bright field imaging, light is scattered by optical heterogeneity of the rhizosphere due to air bubbles and small mismatches in the refractive index of the particles, liquid solution and plant roots. The heterogeneity of the optical properties of the sample creates noise in the image data that strongly limits the ability of algorithms to detect nematodes. As root tissues also produce high levels of biospeckle activity, the exposure time of the camera was adjusted to 100 ms during image acquisition so that the light signal from the root was saturated and changes in light intensity created by nematodes was enhanced. Using this approach, the BSPIM technique was shown to greatly reduce background noise while at the same time enhancing nematode contrast and reducing that from the plant root (Fig. 5). The signal to noise (S/N) ratio was greatly improved. The S/N values for nematodes observed in brightfield images was 7 on average (Standard Error = 3). S/N values determined on BSPIM images were much higher with values averaging 156 (SE = 75). Contrast generated with the biospeckle reconstruction typically increased the S/N ratio by up to 60 times with the S/N value measured in brightfield images of 2 to 21 and the S/N value measured in BSPIM images of 36 to 514 (Wilcoxon signed ranks test, p = 0.028, comparing 6 pairs).

**Figure 6:**
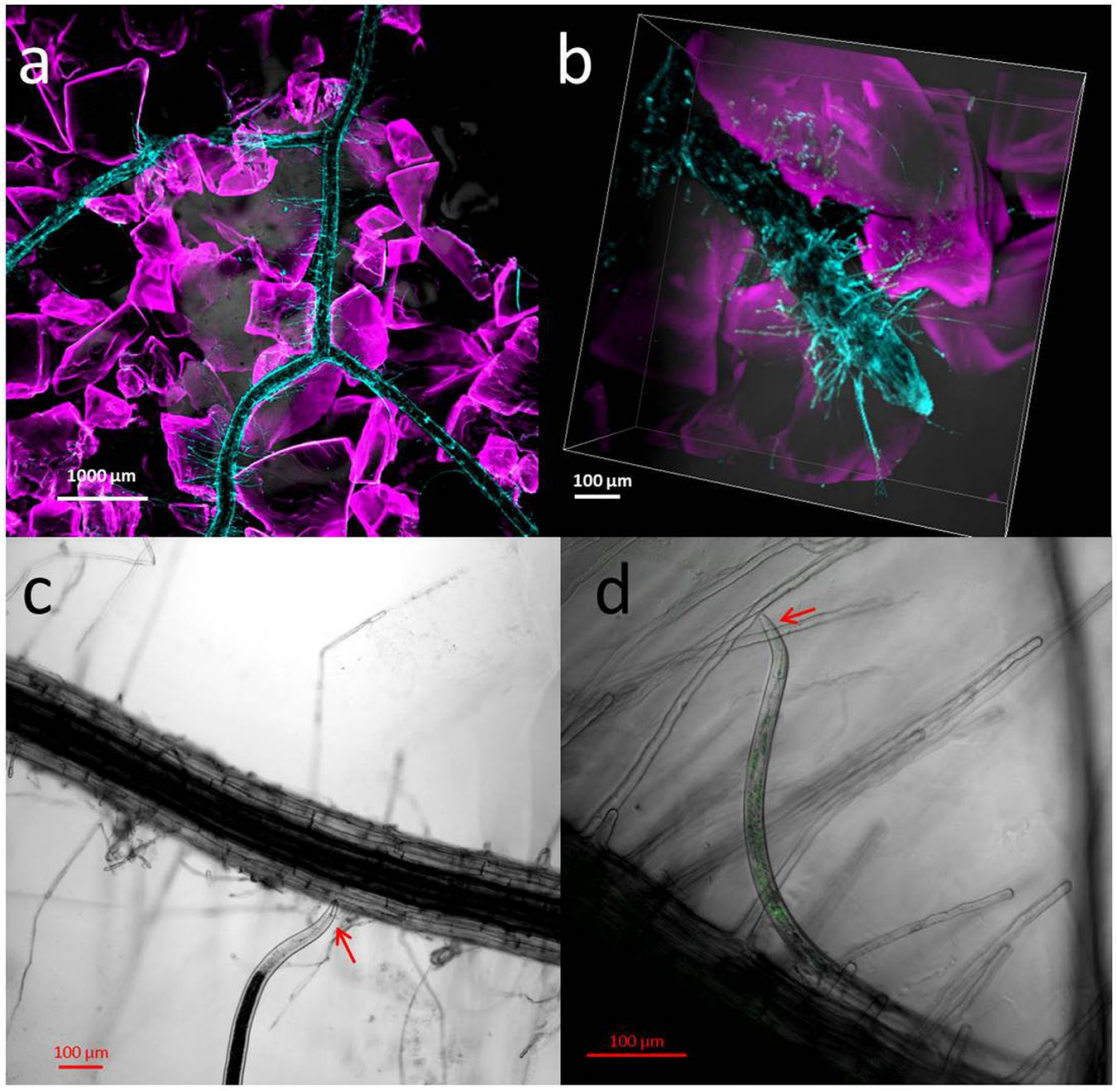
CLSM imaging of plants and nematodes cultured in transparent soil saturated in Ludox® TMA. *N*. *tabacum* (**a**) and *P*. *multiflora* (**b**) stained with calcofluor and growing among transparent soil particles stained with sulforhodamine B. A plant-feeder (**c**) and a bacterivore (**d**) associated with roots of *N*.*tabacum*. Intestinal autofluorescence is visible in green in the bacterivore. Nematode head regions are indicated by red arrows.

Because of the large field of view of our BSPIM system, it can be used to identify regions of interest where subsequent high resolution microscopy can be used to characterise the nematode and its activity in relation to its position on the root. In experiment 4, we showed that CLSM allows high resolution imaging of the nematodes that are detected with BSPIM (Fig. 6). Using CLSM, it was possible to examine the behaviour of the nematode. Observed behaviours included searching and feeding and the displacement of soil particles. Image sequences of plant nematode interactions (Supplementary Figs 5–8 and 3D volume reconstructions were obtained. It was also possible to resolve anatomical features of both roots and nematodes such as root hairs of between 10 to 1000 µm in length and around 10 µm in width and nematode stylets of 20–30 µm in length. It was therefore possible to identify nematodes to functional group and potentially genus and the sites of preferred feeding on or grazing of the plant root.

**Figure 7:**
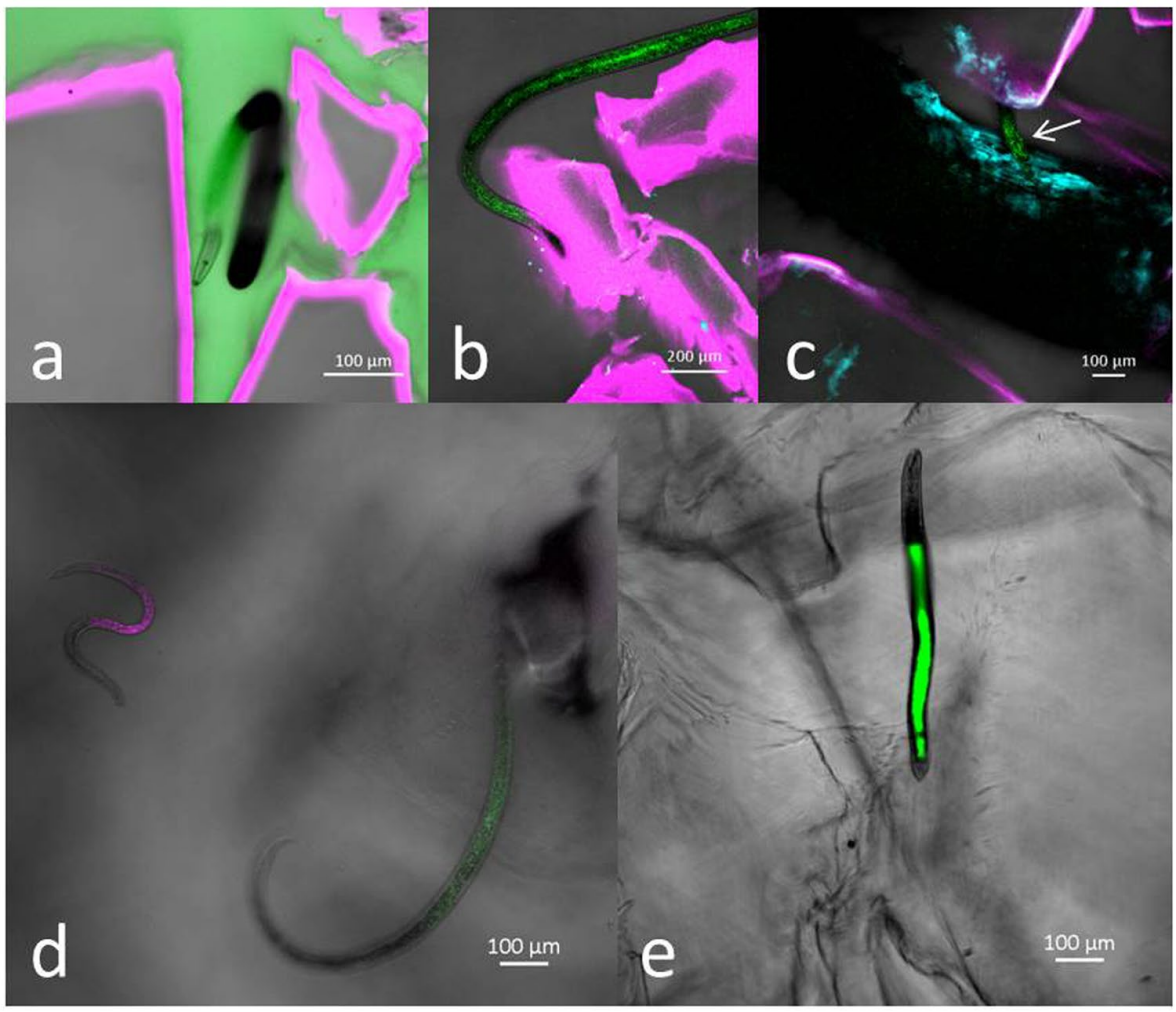
Distinguishing nematodes from their surroundings in transparent soil with CLSM. (**a**) Unstained plant feeder among sulforhodamine B stained particles saturated in Percoll® and 0.1 g L^−1^ calcofluor. (**b**) Autofluorescence in response to 488 nm laser irradiation of a nematode among sulforhodamine B stained transparent soil particles. (**c**) Autofluorescent nematode on calcofluor stained root with sulforhodamine B stained transparent soil particles. The nematode is indicated by the white arrow. (**d**) Intestinal autofluorescence in response to 488 nm (green) and 561 nm (red) laser irradiation. (**e**) Trichodorid with fluorescein in the intestine.

Within the transparent soil microcosm, nematodes provide little contrast to the surrounding medium but this was greatly improved by differential staining. A strong contrast between root structures and their surrounding environment was achieved by staining root cell walls with calcofluor. Transparent soil particles stain easily with a range of dyes for which we used sulforhodamine B (^39^; Fig. 7a–c). In confocal microscopy where laser power and light detection is higher than in light sheet microscopy, autofluorescence of roots and particles was often found to provide sufficient contrast without the need for exogenous stains. Nafion® particle peak emission was obtained with an emission filter of 595 nm and laser excitation of 561 nm, while peak emission of roots was obtained with an emission filter of 515 nm and laser excitation of 488 nm. Autofluorescence was detected in the nematode intestine but at very variable levels (Fig. 7b–d). Intestinal fluorescence was obtained using excitation wavelengths of 488 nm or 561 nm and detection at 515 nm and 595 nm, respectively (Fig. 7d). Better contrast could also be achieved after uptake of a dye by the nematode. This was observed faintly with calcofluor but was stronger with fluorescein (Fig. 7e). The uptake of fluorescein also varied strongly between individuals, being highly visible in some nematodes but absent in most.

## Discussion

Free living nematodes (FLN) have a severe impact on crop production world-wide in terms of yield reduction and concomitant management costs^66^. Nevertheless, the vast majority of FLN species are highly beneficial to soil health and sustainable crop production^67,68^ as part of a trophic web that regulates rates of decomposition and nutrient mineralisation^69–71^ and makes mineralised nitrogen available to plants^72,73^. Traditional approaches to protect crops from root-feeding FLN have, however, often used pesticides which have poor target specificity and are hazardous to environmental safety^74–76^. For example, methyl bromide has had major commercial success because of its broad spectrum efficacy against pathogens, pests and weeds. As with other fumigants, however, the efficacy of methyl bromide arises from its volatility which allows it to travel widely in soil, a property that was later linked to depletion of the ozone layer^77^. Other problems with traditionally used compounds include contamination of soil, drinking water and agricultural produce with substances hazardous to human health, and causing harm to organisms which are not targeted while increasing resistance in those that are^3^. As a result, the focus is now shifting towards designing new management strategies that preserve beneficial contributors to the soil ecosystem, thus safe-guarding long term soil health and crop productivity. However, basic knowledge to design and implement such approaches is lacking due to the wide discrepancy between laboratory and field conditions. For example, testing of new chemical compounds often uses the easily cultured *Caenorhabditis elegans*, which is harmless to plants. Results obtained with such assays are often not readily transferable to herbivorous nematode species and may tend to encourage a non-targeted approach rather than avoid it^74^. Knowledge on nematode trophic groups and their interaction with the environment is also not fully understood^78^ as are even simple patterns of FLN behaviour such as locomotion in soil^12^. It is therefore of great priority to bridge the gap between observations in the laboratory and the field if the targeting of herbivorous FLN is to be improved.

Traditional methods for the study of FLN are based on extraction of nematodes from soil, and subsequent identification and observation of their behaviour using light microscopy. Such quantification is often slow and requires expert knowledge of taxonomy, morphology and behaviour of the nematodes of interest^79^. New developments in DNA amplification, sequencing and barcoding^67,80–82^ have greatly advanced the speed of throughput and precision in identifying soil nematodes. Molecular methods, however, are poorly suited for the investigation of nematode behaviour and responses to environmental stimuli. Many molecular techniques that are available to model organisms such as C. *elegans* e.g. in studying the neuronal basis of behaviour patterns^83–86^ and in imaging gene expression in response to chemical stressors^87–89^, are not relevant to plant-feeding FLN. It is now greatly anticipated that the new generation of live optical imaging technologies will significantly enhance our ability to understand root nematode interactions. Live imaging is achieving far higher resolutions and speed, and allowing deeper imaging within samples^90–92^. However, improving current screens for FLN studies requires specifications that are not achieved by current microscopes. These include (1) label free detection of nematodes extracted from soil, (2) high throughput screening of nematode activity levels, (3) sufficient resolution and field of view for FLN identification and visualisation in the surroundings of the plant.

The present study has addressed many of these limitations using a system that combines light sheet microscopy and biospeckle laser imaging as a new technique- Biospeckle Selective Plane Illumination Microscopy (BSPIM). While Light Sheet Tomography as proposed by Yang, *et al*.^29^ produces images with strong root contrast, nematodes are too difficult to locate due to the heterogeneity of the soil. Unlike imaging systems based on light sheet optics^93–98^, however, contrast in our imaging set-up was created by biospeckle. In the past, biospeckle was used successfully to measure blood flow^99^, detect fungi^30,100^, assess the motility of bacterial colonies^101–103^ and study the responses of root tips to thigmostimuli^44^. Pomarico, *et al*.^104^ used biospeckle to measure the motility of larvae of the pathogenic nematode *Haemonchus contortus*, a parasite in sheep and goats. Our results show that BSPIM is a very powerful approach to detect small levels of biological activity in an optically noisy volume of transparent soil. The technique is fast and importantly enables automated detection of nematode activity. As demonstrated, the present system can be used on nematode communities extracted from soil, without prior knowledge of nematode genotypes and therefore allows monitoring of diverse assemblages whose taxonomy may be unknown. For nematode detection, BSPIM was also more reliable than intestinal autofluorescence, which depends on the presence of heterogeneous materials of different spectral properties^105^, and varies between individuals and genera, with the presence or absence of eggs^106^ as well as age^105^ and diet^105,107^. Our study furthermore showed heat-killed nematodes to display much lower biospeckle compared to live ones and at a level below the threshold used to discriminate live nematodes from background noise. This is a step change compared with conventional methods which try to determine nematode survival via stains, a method which depends on the permeability of the nematode cuticle and the mode of death of the nematode^14,106,108^. In the case of live nematodes the uptake of vital stains can also vary greatly^109^, as was seen in the current study when treatment of nematodes extracted from soil with fluorescein resulted in some individuals but not others becoming stained. Thus this method is unsuitable for both the detection of nematodes and assessment of their survival. Other measures of survival, such as spontaneous movement, although more reliable than staining^106^, serve as proxies for survival rather than measuring biological activity directly. This can be seen in the present study by the slight increase in activity after treatment with trehalose in week 2 compared to week 1 (Fig. 3). BSPIM, however, provides a stain-free, more reliable indicator of nematode survival than conventional methods.

Improving current assays for FLN studies also requires the ability to control the physical environment while monitoring changes in behaviour. At present, *in vitro* tests of treatments for nematode management involve the direct application of diluted chemical compounds to the nematodes, which are then inspected for motility e.g.^110–114^. Such assays do not account for the diffusion of the chemicals through the soil matrix, and sub-lethal behavioural effects. Conventional liquid testing systems also cause nematodes to sink to the bottom of the test well thereby reducing oxygen availability, which may increase stress and therefore mortality rates^115,116^. Recently there has been an effort to move away from homogeneous gels to microfluidic systems that include elements of heterogeneity^117,118^, but these nevertheless lack diversity by consisting of repetitive elements. They are also unsuitable for the culture of plants. Furthermore, in order for high resolution *in vivo* observations to be carried out, these systems require FLN movement to be constrained within an artificial medium^12,119–122^. We have proposed an approach that combines newly developed transparent soils with improved chemistry for biocompatibility of the matching liquid and modular growth chambers for control of soil environmental conditions. Transparent soil is an innovative substrate that mimics the properties of natural soil and which has previously been used for the culture and quantitative imaging of root and microbial processes^39,123^. Using the colloid Ludox® TMA as the refractive index matching agent provided greater transparency than water^124^ with low osmotic potential^39^ at the range of pH observed in soils^125^. We were furthermore able to introduce a soil fumigant, dazomet, into the transparent soil microcosms and subsequently observe its effects on soil-extracted FLN for several weeks. Dazomet, commercially used in Basamid®, is a typical nematicide which releases its active compound (methyl isothiocyanate) in contact with water. By allowing it to dissolve within the refractive index matching liquid, its activation begins on entry into the transparent soil system and without compromising transparency. Transparent soil, therefore, provided realistic growth conditions, with nematodes displaying a range of behaviours such as feeding, and moving between and shifting transparent soil particles, which could be captured in various microscopy set-ups. In our system, pesticides are easily applied and their effects on FLN clearly visible.

Of all the refractive index matching liquids tested, Ludox® TMA was found to be the most suitable medium for the application of BSPIM. When immersed in Ludox® TMA of matching refractive index, transparent soils scatter and absorb light far less than natural soils, providing good conditions for the observation of nematodes and root systems. Scattering and overall transparency was similar when using trehalose, but this had a detrimental effect on nematode survival. Percoll® was also suitable for refractive index matching but its high pH value makes it less optimal for plant growth. The current study used the conventional method of nematode extraction from soil samples by sieving and a modified Baermann funnel method, taking about 2 to 3 days to complete. After this, nematodes were inoculated into microcosms, either individually by hand-picking or as a group in suspension using a syringe. Nematodes can immediately be viewed within the microcosm, although in this study nematodes were usually imaged 24 hours after insertion to allow them time to locate the plant roots. The entire screening process can therefore be completed within 4 days of field samples being taken. While BSPIM would be unsuitable for natural soils, it is anticipated that, following improvements of the image analysis algorithm and optimisation of the acquisition system, the technique can for example be used on diluted soil samples from which coarse soil particles have been removed.

**Figure 8:**
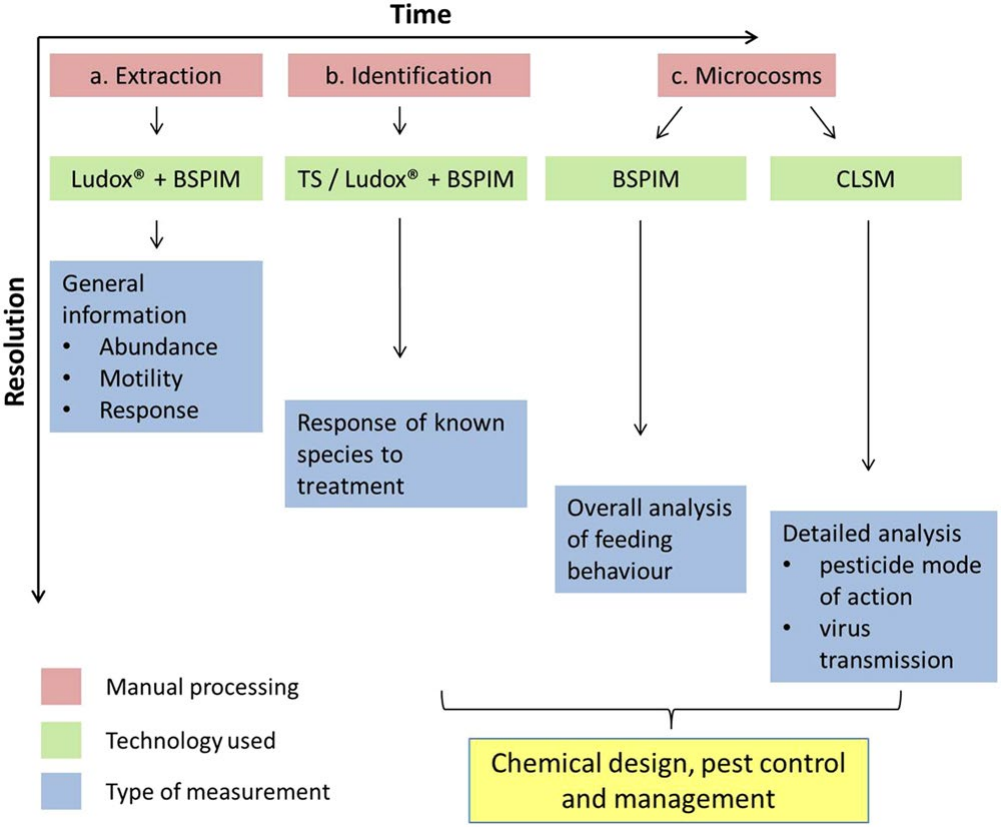
Framework for screening FLN risk to plant health and testing the efficacy of chemical interventions. (**a**) Rapid assessment of nematode population is first required to analyse the threat level. In order to accelerate the analysis, BSPIM can provide basic metrics on the nematode population, providing parameters such as abundance, motility and differential response to treatment. Standardised assays using substrate consisting of Ludox® TMA within a fluorescence cuvette will allow sample preparation and nematode detection in high throughput. (**b**) Once a threat is identified, identification of suitable treatment of the threat can be achieved by initial selection of the FLN of interest and the use of transparent soil assays, for example to test the efficacy and timing of treatment application. (**c**) Finally, detailed studies of nematode behaviour including responses to chemical treatment can be carried using transparent soil microcosms in which nematodes and plants can be co-cultured. Biospeckle (BSL) imaging and Confocal Laser Scanning Microscopy (CLSM) can be combined for efficient quantification of treatment effect on nematode behaviour.

Based on the work presented here, it is possible to propose a framework to accelerate the development of chemical interventions for FLN (Fig. 8). In a first step, rapid assessment of nematode populations extracted from soil is required to analyse the threat level (Fig. 8a), which can then be mapped geographically to the sampling site. In this case, simple, standardised assays can be used based on a substrate of Ludox® TMA within a fluorescence cuvette. Because broad characterisation of nematodes in terms of abundance and activity levels can be obtained in high throughput using BSPIM technology, spatial mapping of threat levels could be obtained within a few days of sampling. Once a threat is identified, suitable treatment can be investigated by selection of the FLN of interest and the use of transparent soil assays and BSPIM detection (Fig. 8b). Finally, detailed studies of effects on behaviour such as feeding or virus transmission can be carried out on nematodes and plants co-cultured in transparent soil microcosms with Biospeckle laser imaging (BSL) and Confocal Laser Scanning Microscopy (CLSM) (Fig. 8c). This framework, while aimed at FLN, could easily be applied to other soil pests such as microbes or fungi, which have already been shown to be detectable by BSL^30,32,126^. Furthermore, the biocompatibility of Ludox® and easy manipulation of chemical properties in terms of nutrients and pH of transparent soil make it a suitable substrate for a wide range of soil-dwelling microorganisms. In summary, the proposed framework significantly accelerates the analysis of nematodes extracted from soils, while the techniques presented here can accelerate the development of more specifically targeted, more ecologically friendly management interventions.

## Electronic supplementary material

Supplementary Information

Supplemental Video

Supplemental Video 1

Supplemental Video 2

Supplemental Video 3

Supplemental Video 4

Supplemental Video 5
