# Supplementary material for: New live screening of plant-nematode interactions in the rhizosphere
Source: Sci Rep. 2018 Jan 23;8:1440. doi: 10.1038/s41598-017-18797-7 (PMC5780396; doi:10.1038/s41598-017-18797-7)
Supplement: Supplementary file 1 — Supplementary Information [file 41598_2017_18797_MOESM1_ESM.pdf]

# Supplementary material

## New live screening of plant-nematode interactions in the rhizosphere

**Authors' names and institutions:**

Felicity E O'Callaghan[1], Roberto A Braga[2], Roy Neilson[1], Stuart A MacFarlane[1], Lionel X Dupuy[1*]

[1]The James Hutton Institute, Invergowrie, Dundee DD2 5DA, Scotland, United Kingdom

[2]Federal University of Lavras, CP 3037 Lavras MG 37.200-000, Brazil

## BSPIM image acquisition and processing:

**Supplementary material S1:** Flowchart detailing the sequence of BSPIM image processing and segmentation

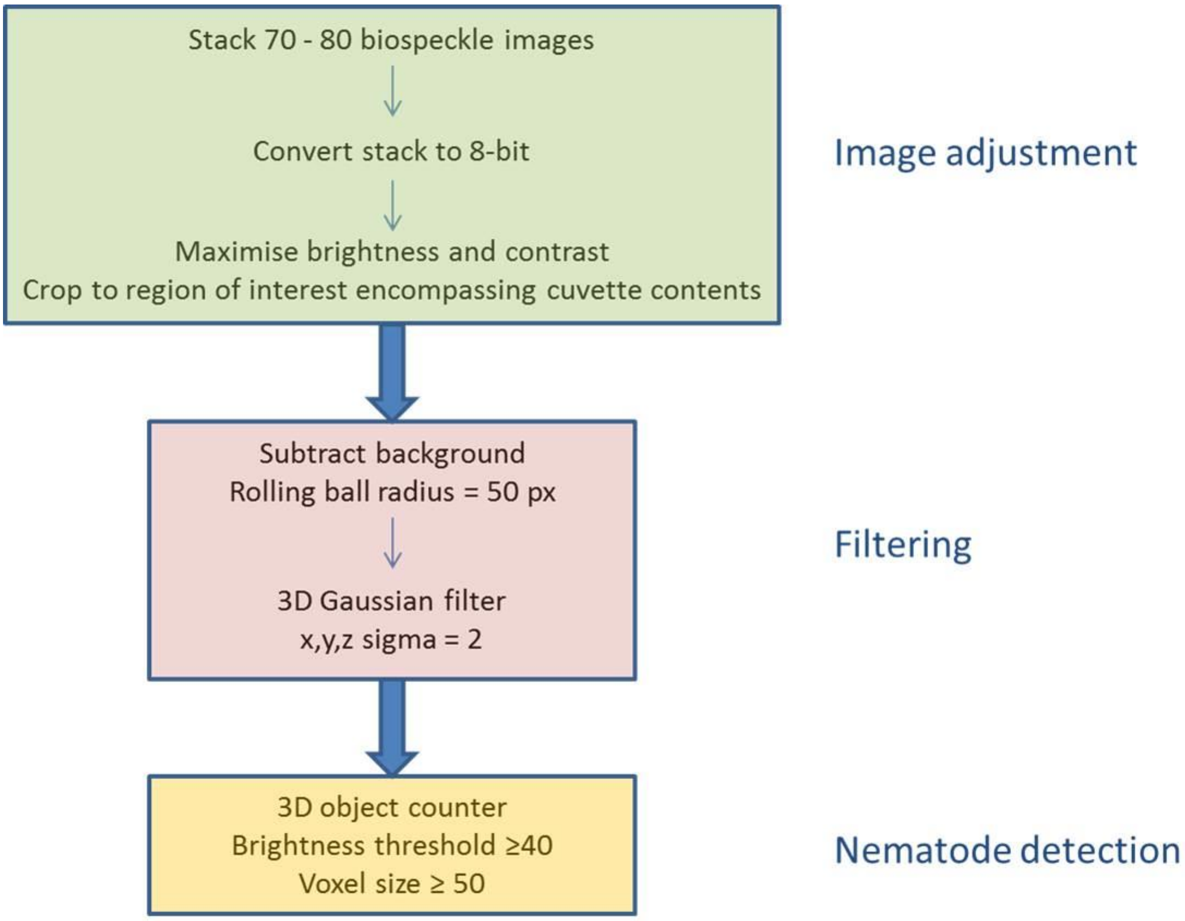

**Supplementary material S2:** ImageJ macroinstruction for the segmentation and detection of nematodes in BSPIM image sequences

```
dir = getDirectory("Choose a Directory ");
list = getFileList(dir);

for (i=0; i <list.length; i++) {
        showProgress(i, list.length);
        list2 = getFileList(""+dir+list[i]);
        run("Image Sequence...", "open=["+ ""+dir+list[i]+list2[0] +"] sort");
        run("BSL SPIM","2");
        }
run("Images to Stack", "name=Stack title=[] use");
//run("Brightness/Contrast...");
setMinAndMax(0, 20);
run("8-bit");
roiManager("Select", 0);
run("Crop");
run("Subtract Background...", "rolling=50 stack");
run("Gaussian Blur 3D...", "x=2 y=2 z=2");
run("3D Objects Counter", "threshold=40 slice=39 min.=50 max.=500 objects statistics summary");
```

**Supplementary video S3:** BSPIM video of nematodes at a boundary of water and Ludox® TMA; 4 nematodes were inserted, 4 objects detected following segmentation. The video consists of a sequence of GD images.

**Supplementary video S4:** BSPIM video of nematodes in transparent soil saturated with Ludox® TMA; 5 nematodes were inserted, 4 objects detected following segmentation. The video consists of a sequence of GD images.

## Confocal timelapse image sequences of FLN behaviour in transparent soil:

**Supplementary video S5:** Nematode interacting with transparent soil particles saturated with Percoll®.

**Supplementary video S6:** Plant feeder among transparent soil particles (stained red with sulforhodamine B) and colloid filled space (shown in green as the fluorescence of calcofluor + Percoll® excited by 488 nm laser).

**Supplementary videos S7 – 8:** FLN among tobacco roots in transparent soil and Ludox® TMA (S7) and Percoll® (S8).
